# IGFBP5 mediates the therapeutic effect of isoliquiritigenin in myocardial ischemia-reperfusion injury via AKT/GLUT4 regulated insulin resistance

**DOI:** 10.3389/fphar.2025.1544869

**Published:** 2025-04-29

**Authors:** Jue Bai, Si-Yuan Yang, Shao-Mei Yu, Ying Cao, Chang-Han Ma, Xuan-Yi Hu, Xiong Chen, Ying-Nan Song, Hong-Jin Chen

**Affiliations:** ^1^ Translational Medicine Research Center, Guizhou Medical University, Guiyang, Guizhou, China; ^2^ Division of cardiac surgery, Guizhou Institute of Precision Medicine, Affiliated Hospital of Guizhou Medical University, Guiyang, Guizhou, China; ^3^ Department of Ultrasound Medicine, The Affiliated Hospital of Guizhou Medical University, Guiyang, Guizhou, China; ^4^ Department of Anesthesiology, The Affliated JinYang Hospital of Guizhou Medical University, The Second People’s Hospital of Guiyang, Guiyang, Guizhou, China; ^5^ Department of Endocrinology, the First Affiliated Hospital of Wenzhou Medical University, Wenzhou, China; ^6^ Department of Pharmacology, School of Basic Medical Sciences, Guizhou Medical University, Guiyang, Guizhou, China

**Keywords:** IGFBP5, isoliquiritigenin, myocardial ischemia/reperfusion injury, insulin resistance, GLUT4

## Abstract

**Background:**

Myocardial ischemia/reperfusion injury (MIRI) is a critical problem in cardiovascular medicine, often occurring after coronary revascularization procedures or cardiopulmonary bypass. The characters of MIRI are both energy metabolism disturbances and severe myocardium insulin resistance (IR), which exacerbated myocardial damage and cell death. Isoliquiritigenin (ISL), a flavonoid derived from licorice roots (*Glycyrrhiza* spp.), has demonstrated protective effects on MIRI. However, the potential cardio-protective effects and mechanism of ISL in MIRI remain unclear.

**Propose:**

In this study, we aimed to investigate ISL’s therapeutic effects on MIRI. Moreover, we elucidate the underlying mechanisms of ISL regulated myocardium insulin resistance *in vivo* and *in vitro*.

**Methods:**

*In vivo,* SD rats underwent left anterior descending coronary artery ligation/reperfusion to induce MIRI. Chest echocardiography was performed to monitor cardiac function post-reperfusion, followed by measurement of myocardial injury and IR markers. *In vitro,* H9C2 cardiomyocytes subjected to oxygen-glucose deprivation/reperfusion (OGD/R). Markers associated with myocardial injury and IR were assessed. Then, we identified potential therapeutic targets IGFBP5 for MIRI by network pharmacology and molecular docking analysis. Finally, lentivirus were used to silence or over-express IGFBP5 to elucidate the role of IGFBP5 in regulating the therapeutic effects of ISL on IR in MIRI.

**Results:**

In the present study, *In vivo* experiments demonstrated that ISL attenuated myocardial infarct size, decreased serum markers of myocardial injury, improved left ventricular systolic function, and enhanced insulin sensitivity. *In vitro* data revealed that ISL ameliorated glucose uptake and cell survival rate. Furthermore, ISL increased AKT phosphorylation and upregulated membrane-bound GLUT4 (M-GLUT4) protein expression levels. These effects of ISL are mediated by the induction of IGFBP5, as demonstrated using gene-specific shRNA or overexpression for IGFBP5.

**Conclusion:**

Our results reveal that ISL protects against myocardial damage caused by MIRI through the regulation of IR via the IGFBP5/AKT/GLUT4 pathway.

## Graphical Abstract

**Figure:**
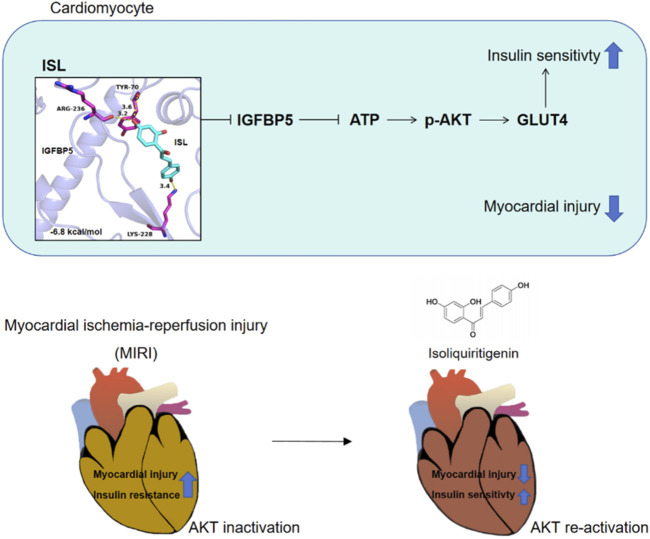

## 1 Introduction

Acute myocardial infarction (AMI) occurs due to severe and persistent myocardial ischemia, leading to irreversible tissue damage. The most common underlying cause is plaque rupture or erosion in the epicardial coronary arteries, resulting in superimposed thrombosis and arterial occlusion (Ibánez et al., 2017). These changes can culminate in heart failure, potentially fatal arrhythmias, and remain a leading cause of death worldwide (Thygesen et al., 2018). To date, successful and prompt myocardial reperfusion has become the most effective intervention to limit infarct size and improve patient outcomes (Reed et al., 2017; Anderson and Morrow, 2017). Although reperfusion has the potential to salvage ischemic myocardium after infarction, it can paradoxically worsen and accelerate myocardial injury, a phenomenon known as myocardial ischemia/reperfusion injury (MIRI) (Veltman et al., 2021). Despite extensive studies on MIRI treatment, effective therapeutic drugs remain an unmet clinical need.

A complex interplay of pathological processes underlies MIRI, such as autophagy, apoptosis, pyroptosis, immune system activation, mitochondrial dysfunction, inflammation, and oxidative stress (Heusch, 2020; Wu et al., 2018). Previous studies identified insulin resistance (IR) as a key initiator of MIRI, with glucose transporter 4 (GLUT4) potentially playing a critical role (Liang et al., 2011; Liang et al., 2008). IR signifies a diminished cellular responsiveness to insulin. However, insulin stimulates the translocation of intracellular GLUT4 vesicles to the plasma membrane via exocytosis, facilitating glucose uptake and transportation (Hesselink et al., 2016; Klip et al., 2019). Furthermore, dysregulation of the protein kinase B/AKT signaling pathway significantly impacts IR. Impaired AKT activity leads to decreased GLUT4-mediated glucose uptake in skeletal muscle and adipose tissue (Williamson and Sheedy, 2020; Ishiki and Klip, 2005). Therefore, regulating AKT signaling to enhance insulin sensitivity presents a promising therapeutic strategy for MIRI.

**FIGURE 1:**
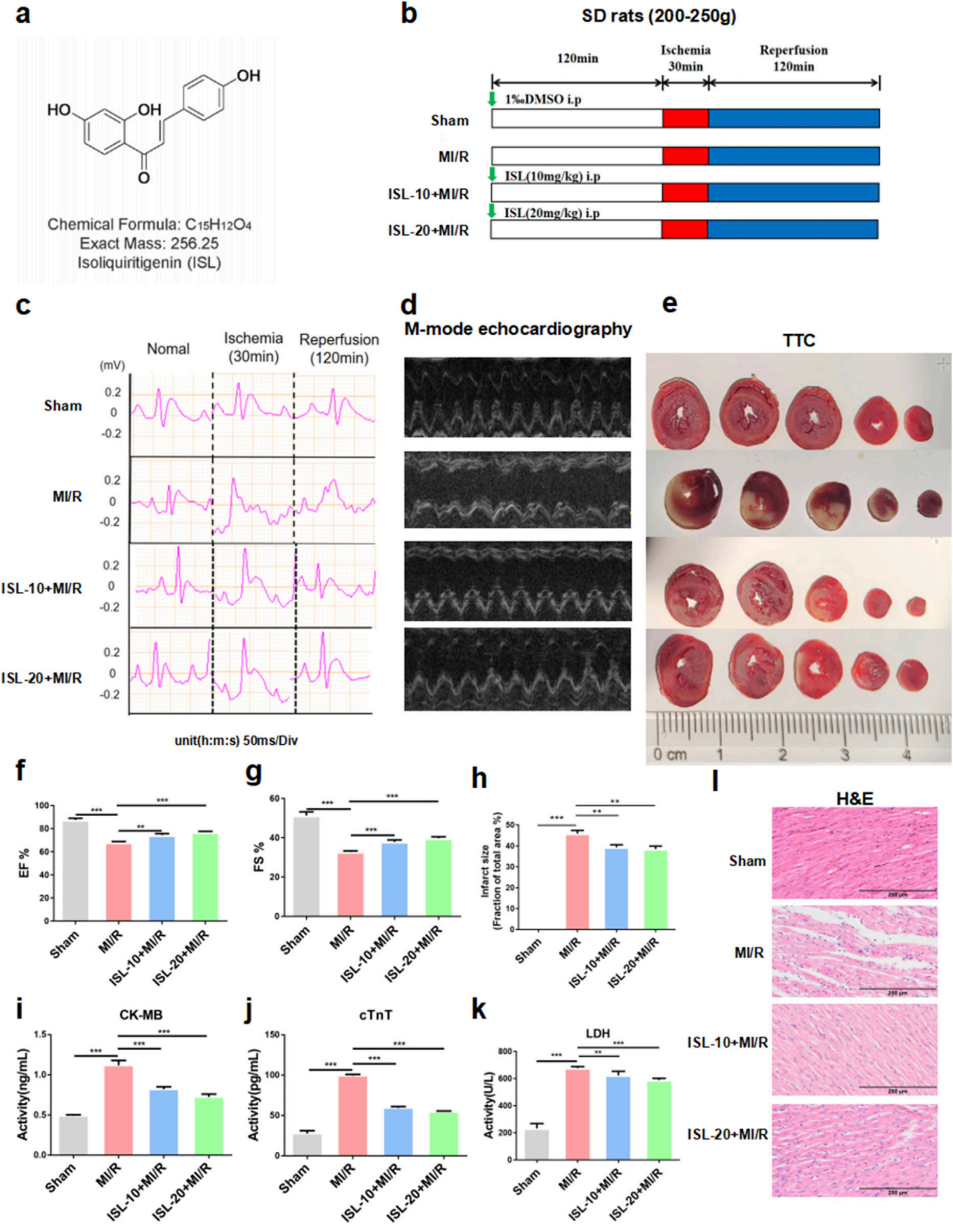
The protocol of this experiment and ISL attenuated MIRI in rats. **(a)** The molecular structure of ISL. **(b)** Experimental protocol of ISL administration on MI/R injury in rat. **(c)** Representative ECG traces in each group. **(d)** M-mode echocardiography performed 2 h after MI/R injury in a rat study (n = 5). **(e)** TTC staining detected the myocardial infarction area (n = 3). **(f,g)** The EF% and FS% of M-mode echocardiography. **(h)** The quantification of infarct size was displayed. **(i–k)** The levels of CK-MB, cTnT and LDH were detected (n = 5). (L) H&E staining of myocardial tissue. Scale bar: 200 μm *p < 0.05, ***p < 0.001.

In recent decades, plant-derived compounds have garnered significant interest in treating various life-threatening illnesses. Isoliquiritigenin (ISL, Figure 1a), a chalcone flavonoid isolated from licorice roots (*Glycyrrhiza* spp.), exhibits a range of biological and pharmacological properties, including anti-inflammatory and anti-oxidant effects (Huang et al., 2020). Recent studies have demonstrated that ISL protects against diabetic cardiomyopathy and AMI (Gu et al., 2020; Yao et al., 2022). Additionally, ISL significantly reduces IR levels in a diet-induced obesity model of C57BL/6J mice (Lee et al., 2018). Moreover, its efficacy against MIRI has been testified (Yao et al., 2024; Shen et al., 2024). However, the molecular mechanism of ISL therapy of MIRI is still unclear, and the target of ISL also needs to explore. In this study, we investigated the protective effects of ISL on MIRI *in vivo* and *in vitro*. Then, using docking analysis, we explored that ISL could target insulin-like growth factor binding protein 5 (IGFBP5) to activate AKT/GLUT4 pathway which attenuated myocardial IR. These results reveal the possibility of ISL as a potential drug for the treatment of MIRI.

## 2 Materials and methods

### 2.1 Materials

ISL (Aladdin, Shanghai, China, Figure 1a) was dissolved in dimethyl sulfoxide (DMSO; Solarbio, Beijing, China) and saline (0.9% NaCl) for *in vitro* and *in vivo* experiments, respectively.

### 2.2 Animals and treatment

SD rats weighed 200–250 g were obtained from the Animal Center of Guizhou Medical University (Guiyang, China). The Ethics Committee at Guizhou Medical University approved the study protocol (No.2200398). All rats were housed under controlled conditions (temperature-controlled room, 50% humidity, 12-h light/dark cycle) with *ad libitum* access to standard water and food. Before myocardial ischemia-reperfusion (MI/R) surgery, rats were randomized into four groups and received daily intraperitoneal injections of either vehicle (DMSO in normal saline), ISL (10 mg/kg), or ISL (20 mg/kg) for 3 days. The groups were designated as follows: (1) Sham group: sham surgery, n = 8 (2) MI/R surgery: myocardial ischemia/reperfusion surgery, n = 8 (3) ISL-10+MI/R: 10 mg/kg ISL administered before MI/R surgery, n = 8 and (4) ISL-20+MI/R: 20 mg/kg ISL administered before MI/R surgery, n = 8.

### 2.3 MI/R surgery

Two hours after the final treatment on day 3, anesthesia was induced with intraperitoneal tribromoethanol (10 mg/kg). Animals were mechanically ventilated with room air using a small animal respirator set at a 1:1 inspiratory-to-expiratory ratio, tidal volume of 4 mL, and respiratory rate of 80 breaths/min. Electrocardiogram (ECG) electrodes were attached to the limbs for continuous monitoring throughout the surgery (MD3000, Zhenghua Biological Instrument Equipment Co., Ltd., Anhui, China). The surgical area on the chest was shaved and sterilized. A left thoracotomy was performed by dissecting the pectoralis major muscle layer-by-layer with hemostatic forceps. After exposing the left atrial region, 3–4 ribs were carefully divided on the left side of the sternum to access the heart. The pericardium was dissected using ophthalmic forceps to visualize the left anterior descending (LAD) coronary artery. Myocardial ischemia was induced by transient occlusion of the LAD coronary artery using a 6–0 silk suture. Electrocardiography was continuously monitored. Successful LAD occlusion was confirmed by sustained ST-segment elevation >15 min and epicardial cyanosis distal to the ligature. After 30 min of ischemia, the occlude (polyethylene tubing) was removed to achieve reperfusion. Sham-operated animals underwent identical procedures except for LAD occlusion. Following reperfusion, rats were anesthetized and euthanized with CO_2_ inhalation. Heart tissue and blood samples were collected for further analysis.

### 2.4 Treatment and culture of cells

H9C2 cells, acquired from the National Collection of Authenticated Cell Cultures (Shanghai, China), were cultured at 37°C with 5% CO_2_ with Dulbecco’s modified Eagle’s medium (DMEM; Gibco, United States) supplemented with 5.5 mM D-glucose, 10% fetal bovine serum, 100 U/mL penicillin-streptomycin solution in a humidified incubator. Confluent H9C2 cells were pre-treated for 2 h with different concentrations of commercially available ISL in DMEM before exposure to oxygen-glucose deprivation/reperfusion (OGD/R). Untreated control cells received an equivalent volume of DMSO to mimic the treatment group’s solvent exposure. Using lentivirus, cells were targeted to silencing IGFBP5 (shIGFBP5#1: 5′-ACG​GCG​AGC​AAA​CCA​AGA​TAG-3′, shIGFBP5#2: 5′-CTG​GGC​CTC​TTT​CGT​GCA​TTG​T-3′, and shIGFBP5#3: 5′-GTG​AAG​AAG​GAT​CGC​AGA​AAG-3′) or overexpressing IGFBP5, along with match negative control (GenePharma, Shanghai, China). After 72 h post-transfection, western blotting was used to assessed the transfection efficiency.

### 2.5 AAV9 injection

AAV9 vectors packaging sh-IGFBP5 (5′-CTG​GGC​CTC​TTT​CGT​GCA​TTG​T-3′) or oe-IGFBP5 were prepared by GenePharma (Shanghai, China). Though tail vein, one rat had been injected 3 × 10^11^ virus vector genomes. After 3 weeks, all rats underwent MI/R surgery. Then, cardiac tissues were collected for further analysis.

### 2.6 OGD/R cell model and group allocation

H9C2 cells were cultured in glucose-free DMEM (Gibco, United States) for 3 h under hypoxic conditions (37°C, 1% O_2_ and 5% CO_2_). Subsequently, the medium was replaced with normal DMEM, and cells were incubated for an additional 30 min under normoxic conditions. Cells were randomly allocated into four groups: control, OGD/R, and two additional groups treated with different concentrations of ISL (10 µM or 20 µM).

### 2.7 Assay for cell viability

H9C2 cell viability was assessed by CCK-8 (K1018, Apexbio, Houston, TX, United States). H9C2 cells were seeded at a density of 8,000 cells per well in 96-well plates. After incubation with different concentration of ISL for 24h, 10 μL of CCK-8 solution was added to each well. After incubated for an additional 2 h, the absorbance was measured at 450 nm using a microplate spectrophotometer.

### 2.8 Measurement of ATP,LDH,CK-MB and cTnT

Adenosine 5′-triphosphate (ATP, G0857W, Grace Biotechnology, Suzhou, China) and lactate dehydrogenase (LDH, A020-2, Nanjing Jiancheng Institute of Bioengineering, Nanjing, China) were measured by commercially available kits. ELISA kits (ZCIBIO, Shanghai, China) were used to determine cTNT (#ZC-37374) and CK-MB (#ZC-36768).

### 2.9 Echocardiographic assessment

After 2 h reperfusion, left ventricular (LV) function was testified by a high-resolution small animal ultrasound imaging system (E5, SonoScape, Shenzhen, China). The fully anesthetized rat was secured in a supine position on a heated platform to maintain body temperature during image acquisition. An appropriate amount of chelator was applied to the rat’s chest for optimal echocardiographic signal transmission. Left ventricular function was assessed in M-mode, with the following indices measured: left ventricular ejection fraction (EF) and left ventricular fractional shortening (FS).

### 2.10 Glucose tolerance tests

Following the removal of the plastic tube, a 2 g/kg glucose solution was administered intraperitoneally to the rats. Blood glucose levels were measured at baseline (immediately before injection) and at 30, 60, and 120 min post-injection. Blood samples were collected from the tip tail using a standardized technique. Blood glucose concentration was determined at each time point using a glucometer (Yuwell, Jiangsu, China).

### 2.11 Insulin tolerance tests

After removal of the plastic tube, the rats received an intraperitoneal injection of insulin (1.5 U/kg). Blood samples were collected and measured from the tip tail at baseline and 30, 60, and 120 min post-injection.

### 2.12 Assessment of myocardial infarction

Hearts were excised and frozen at ˗ 80°C for 12 min. Subsequently, frozen hearts were sectioned into 1.5 mm-thick transverse slices and incubated with 2% 2,3,5-triphenyl tetrazolium chloride (TTC, G3005, Solarbio, Beijing, China) for 30 min at 37°C under physiological conditions (pH 7.4). Infarct size was quantified using photomicrographs and Image-ProPlus 6.0 software (Media Cybernetics Company, MD, United States). Myocardial infarct rate (%) was calculated as the ratio of the infarct area (unstained white tissue) to the total cross-sectional area of the myocardium, multiplied by 100%.

### 2.13 Hematoxylin & Eosin (H&E) staining

Heart tissue was fixed in 4% paraformaldehyde, dehydrated using an automated dehydrating apparatus (HP300, Dakewe, Shenzhen, China), and embedded in paraffin. Subsequently, 7 mm sections were obtained using a manual sectioning machine (HistoCore BIOCUT, Leica, Germany). These sections were stained using H&E and visualized under a light microscope for imaging.

### 2.14 Immunocytofluorescence staining

After fixed with 4% paraformaldehyde for 10 min, H9C2 cells were permeabilized using 0.3% Triton X-100. Subsequently, cells were incubated with anti-GLUT4 antibody (1:400, 66846-1-Ig, Proteintech Group, Rosemount, IL, United States) overnight at 4°C. Following washes with PBS, cells were incubated with CY3-conjugated secondary antibody (SA00009-1, Proteintech Group) for 1 h at room temperature in the dark. Then, 4′,6-diamidino-2-phenylindole (Solarbio, Beijing, China) was visualized the nuclei. Images were captured via a fluorescence microscope (SpinSR10, Olympus, Tokyo, Japan). Data were obtained from at least three independent replicates.

### 2.15 Glucose uptake measurement

Glucose uptake was quantified using a commercially available fluorescent glucose probe 2-(N-(7-Nitrobenz-2-oxa-1, 3-diazol-4-yl) amino)-2-deoxyglucose (2-NBDG; HY-116215, MedChemExpress, Shanghai, China). Cells were washed with warm (37°C) PBS and then incubated for 20 min at 37°C. To minimize metabolic activity before fluorescence intensity measurement by flow cytometry (B90883, Beckman Coulter, Brea, CA, United States), cells were washed again with cold (4°C) PBS.

### 2.16 Network pharmacology

The molecular structure of ISL was validated through the PubChem chemical database (https://pubchem.ncbi.nlm.nih.gov), from which the canonical SMILES identifier was retrieved. Putative pharmacological targets of ISL were predicted using the SwissTargetPrediction platform (https://swisstargetprediction.ch) with a probability threshold >0 for preliminary screening (Daina et al., 2019). Concurrently, disease-associated targets related to MIRI were acquired from the GeneCards database (https://www.genecards.org) by searching the key term “Myocardial ischemia reperfusion injury”, followed by ranking based on relevance scores and selection of targets meeting the established cutoff criterion (Score ≥5). Through intersection analysis of the compound-target and disease-target datasets using a Venn diagram approach, 49 overlapping targets were identified as potential therapeutic targets for ISL in MIRI intervention.

### 2.17 Visualization and enrichment analysis of target related proteins

Utilized the STRING database to obtain protein genes related to potential targets, and analyzed the interaction information, constructed PPI network. Metascape database was investigated for GO and KEGG enrichment analysis.

### 2.18 Molecular docking

**FIGURE 4:**
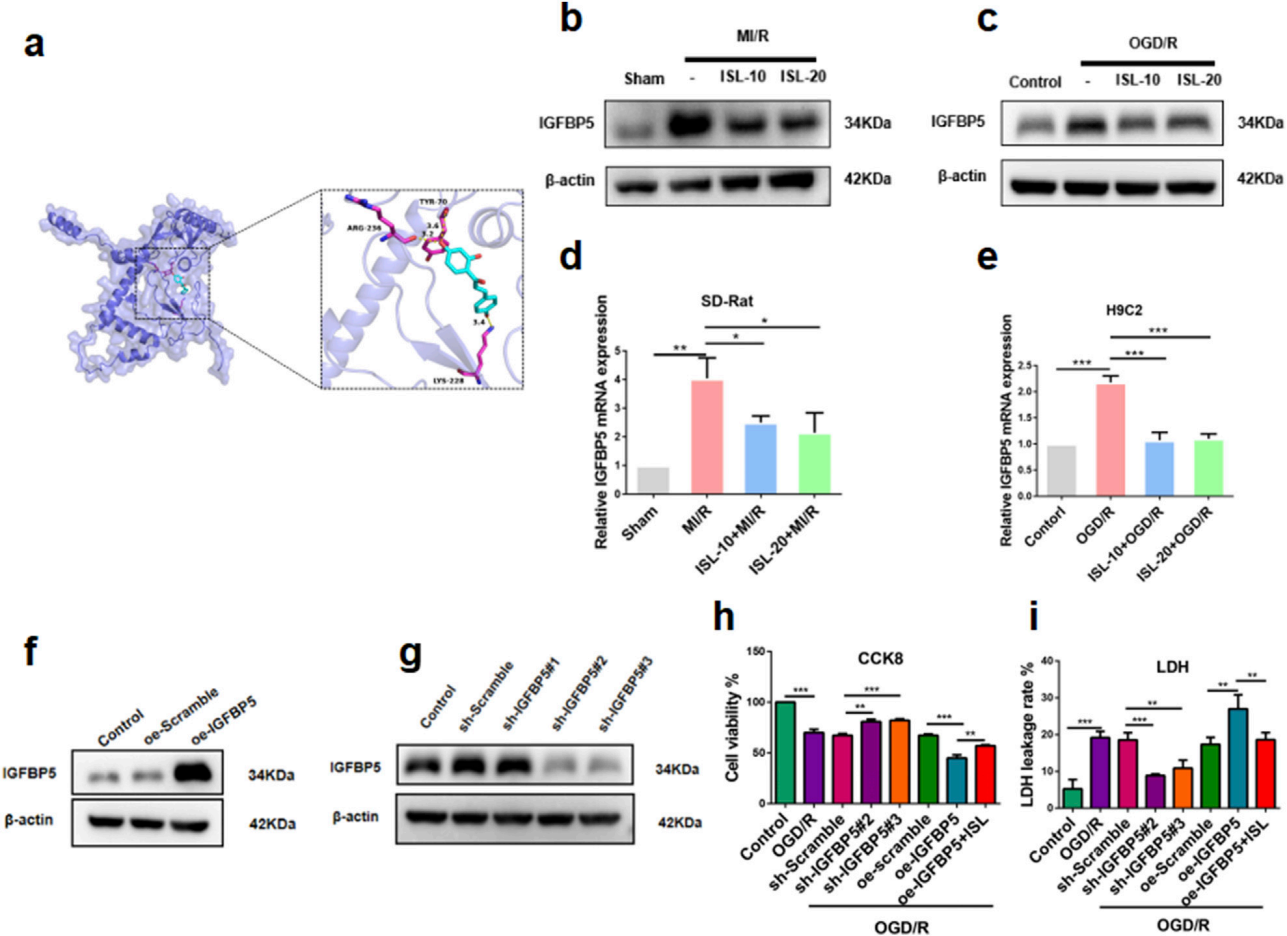
Functional analysis of ISL against MIRI. **(a)** Molecular docking of ISL with IGFBP5. **(b–e)** The protein **(b–c)** or mRNA expression levels **(d,e)** of IGFBP5 in heart tissues and H9C2 cells. **(f,g)** Western blotting analysis of oe-IGFBP5 or sh-IGFBP5 changed IGFBP5 expression in H9C2 cells. **(h,i)** CCK8 assay **(h)** and LDH leakage rate **(i)** was testified the H9C2 cells viability with lentivirus interference or ISL treatment. **p < 0.01, **p < 0.01, ***p < 0.001.

According to previous studies of Open Babel and AutoDock Vina (1.2.0), the optimal pose was selected for analysis about the interaction between of ISL and IGFBP5. Additional, using the PyMOL software, IGFBP5-ISL interaction figure was exhibited (Figure 4a). A slate cartoon model was used to represented IGFBP5 protein. ISL is represented as a cyan stick, and IGFBP5-ISL binding site is represented as a magenta stick structure. Hydrogen bonds were shown at yellow dashed lines; ionic interactions were indicated by magenta dashed lines; and hydrophobic interactions were exhibited by green dashed lines (O'Boyle et al., 2011; Eberhardt et al., 2021; Trott and Olson, 2010).

### 2.19 Western blot

Total protein lysates were prepared by homogenizing tissues or cells in pre-cooled radio-immunoprecipitation assay buffer (R0010, Solarbio, Beijing, China) at 4°C for 30 min. Samples were supplemented with 5X protein loading buffer and boiled for 10 min to achieve complete denaturation. For the detection of GLUT4, membrane proteins were extracted using a membrane protein extraction kit (BB-3103, Beatbio, Shanghai, China). Equal amounts of total protein (25–30 μg) were electrophoresed on SDS-polyacrylamide gel. Proteins were transferred to a polyvinylidene difluoride membrane (PVDF, Millipore, United States). Membranes were blocked with 3% non-fat dry milk (1172GR500, BioFroxx, Germany) or bovine serum albumin (BSA) for 1 h at room temperature. Then, the membranes incubated at 4°C overnight with the primary antibodies, including GLUT4 (1:1000, 66846-1-Ig, Proteintech), phosphorylated-AKT (p-AKT) (1:1000, AF0016, Affinity), AKT (1:1000, 51077-1-AP, Proteintech), IGFBP5 (1:1000, ab254324, Abcam), β-actin (1:1000, 20536-1-AP, Proteintech), and Na^+^/K^+^-ATPase (1:1000, ab7671, Abcam). After washing with Tris-buffered saline Tween 20, membranes were incubated with goat anti-mouse or anti-rabbit secondary antibodies for 1 h at room temperature and visualized using enhanced chemiluminescence reagents (Bio-Rad, CA, United States). Protein levels were quantified using ImageJ software (version 1.53a, Bethesda, United States) and normalized to their respective loading controls (β-actin and Na^+^/K^+^-ATPase) for whole cell or tissue lysates and membrane fractions.

### 2.20 Statistical analysis

Data are presented as mean ± standard errors of the mean. Differences among multiple groups were compared using a one-way analysis of variance. Statistically significant were been considered at *P-values < 0.05*. Statistical analyses were performed using GraphPad Prism 8 (San Diego, CA, United States).

## 3 Results

### 3.1 ISL protects against MIRI in rats

We investigated the protective effects of ISL against MIRI *in vivo* using a rat model. Rats received ISL for 3 days followed by 30 min of ischemia and 2 h of reperfusion (Figure 1b). ECG was used to assess cardiac function after MIRI. A sustained ST-segment elevation on ECG is a marker of cardiac ischemia. Notably, the ST-segment continuously increased during ischemia and remained elevated after reperfusion, gradually returning to baseline, indicating the successful establishment of the MI/R model. Moreover, MI/R rats exhibited a significantly larger infarct size compared to the sham group. However, pre-treatment with ISL significantly reduced ST-segment elevation and infarct size compared to the MI/R group (p = 0.0004, Figures 1c,e,h). Left ventricular M-mode echocardiography revealed restored cardiac function in the ISL + MI/R group compared to the MI/R group (ISL-10+MI/R *v. s.* MI/R, p = 0.0047; ISL-20+MI/R *v. s.* MI/R, p = 0.0038). And pre-treatment with ISL significantly attenuated MI/R-induced reductions in ejection fraction and fractional shortening (Figures 1d,f,g). Serum levels of myocardial injury markers, like CK-MB, cTnT and LDH were significantly elevated in the MI/R group compared to the sham group (p < 0.0001). ISL significantly reduced these elevated markers, indicating its protective effect (p < 0.0001, Figures 1i–k). H&E staining revealed varying degrees of myocardial damage across groups. Myocytes in the sham group appeared normal, with no signs of hemorrhage or neutrophilic infiltration. Conversely, the MI/R group exhibited severe damage to the myocardium in the ischemic area, characterized by disintegrated myocardial fibers and inflammatory cell infiltration, particularly neutrophils. Pretreatment with ISL significantly attenuated these MI/R-induced injuries (Figure 1l).

### 3.2 ISL protects against IR in MIRI via the AKT/GLUT4 pathway

**FIGURE 2:**
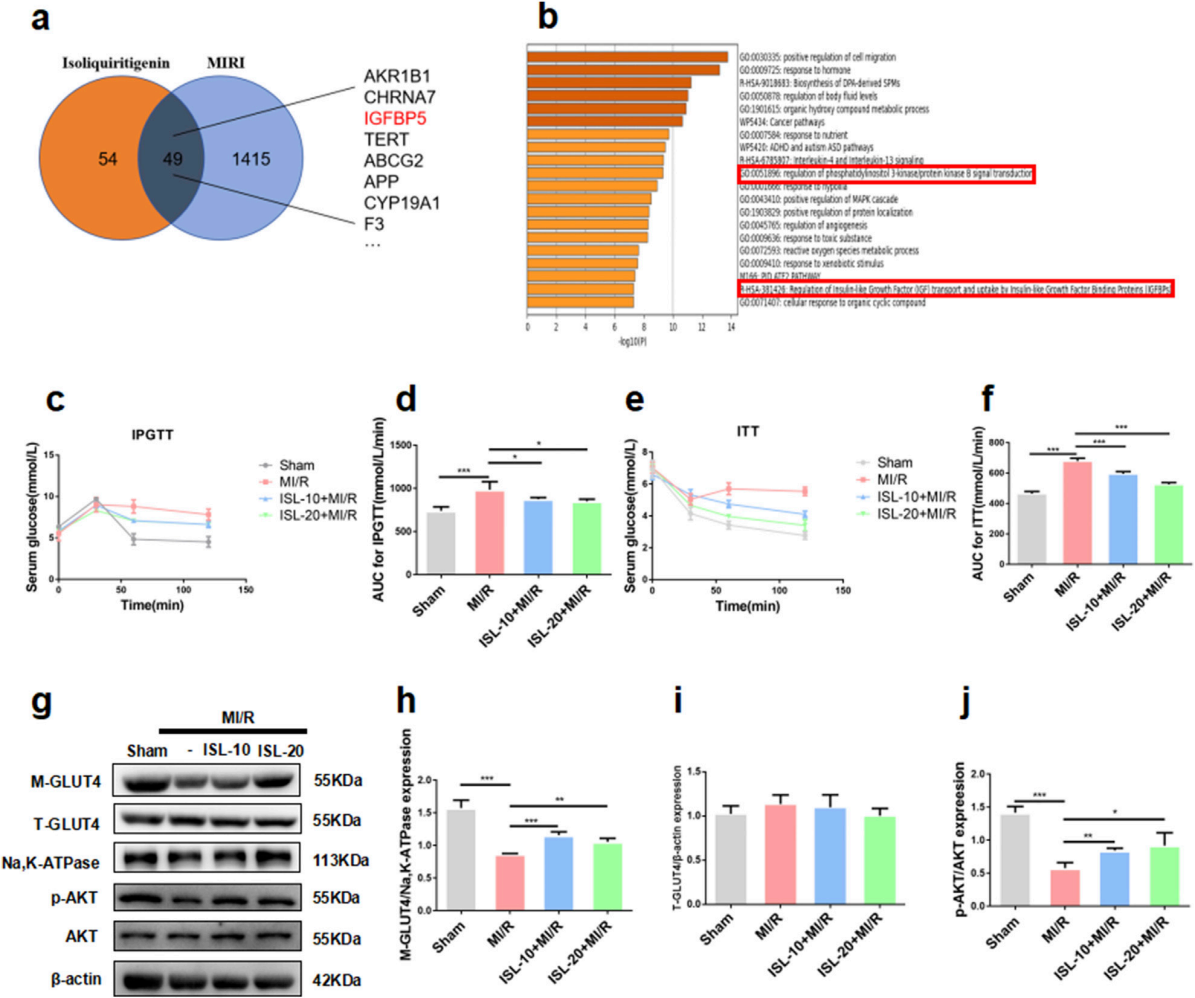
ISL attenuated IR by AKT/GLUT4 pathway to improve MI/R injury. **(a)** Venn diagram of ISL-targeted genes and MI/R injury-targeted genes. **(b)** GO and KEGG enrichment of the 49 merge genes. **(c–f)** IPGTT and ITT was performed after the re-perfusion begin of MI/R. The area under the curve (AUC) of IPGTT **(d)** and ITT **(f)** (n = 5). **(g–j)** The representative western blot bands and quantitation of M-GLUT4, T-GLUT4, p-AKT, and AKT in rat myocardial tissue. *p < 0.05, **p < 0.01, ***p < 0.001.

To identify potential therapeutic targets of ISL for MIRI, we analyzed the SwissTargetPrediction and GeneCards database. This analysis revealed 49 overlapping targets between ISL and those associated with MIRI (Figure 2a; Supplementary Table S1). GO and KEGG analysis of these proteins suggested that the ISL treatment for MIRI was closely associated with ‘Reaulation of Insulin-like Growth Factor (lGF) transport and uptake by Insulin-like Growth Factor Binding Proteins (lGFBPs) (Figure 2b). Our previous study showed that MIRI patients have serious glucose abnormal (Chen et al., 2023). In order to investigate that whether ISL attenuates MIRI via glucose metabolism. Then, the impaired glucose tolerance test (IPGTT) and insulin tolerance test (ITT) had been testified *in vivo*. As shown in Figures 2c–f, ISL administration significantly induced higher blood glucose levels and area under the concentration-time curve compared to the MI/R group (IPGTT AUC, Sham *v. s.* MI/R p = 0.0007; ISL-10+MI/R *v. s .*MI/R p = 0.0311; ISL-20+MI/R *v. s.* MI/R, p = 0.0133) (ITT AUC, Sham *v. s.* MI/R, p < 0.0001; ISL-10+MI/R *v. s.* MI/R,p < 0.0001; ISL-20+MI/R *v. s.* MI/R, p < 0.0001), which suggesting its potential role in regulating insulin sensitivity. In addition, the KEGG analysis showed that PI3K pathway was enrichment for the 49 genes (Figure 2b). Lots studies show that activation of AKT, the mainly target gene of PI3K pathway, could promote GLUT4 translocation and attenuates IR (Yang et al., 2024). Figures 2g–j testified that MI/R group decreased p-AKT (p = 0.0003) and M-GLUT4 (p = 0.0005) expression, whereas pretreatment with ISL significantly increased p-AKT (ISL-10+MI/R *v. s.* MI/R p = 0.0072; ISL-20+MI/R *v. s.* MI/R, p = 0.0486) and M-GLUT4 (ISL-10+MI/R *v. s.* MI/R p = 0.0007; ISL-20+MI/R *v. s.* MI/R, p = 0.004) protein levels. Overall, these data potential investigated that ISL improves IR in MIRI via AKT/GLUT4 pathway.

### 3.3 ISL attenuates OGD/R-induced cell death and IR in H9C2 cells

**FIGURE 3:**
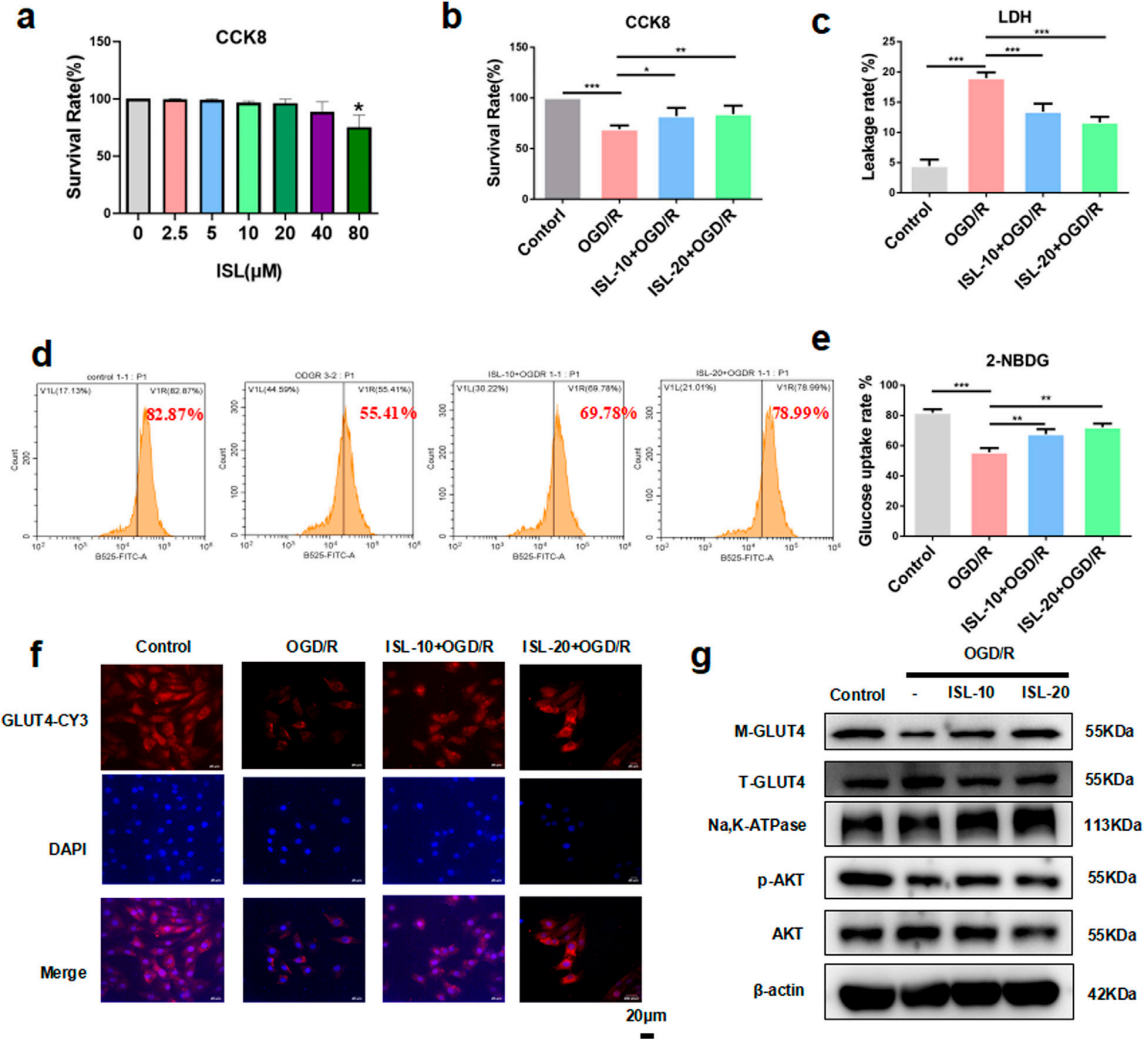
ISL attenuated OGD/R-induced cell death and insulin resistance in H9C2 cells. **(a)** CCK8 assay detected the H9C2 cells viability with or without ISL treatment. **(b)** CCK8 assay detected the effects of ISL on OGD/R injury in H9C2 cells. **(c)** The levels of LDH leakage rate were detected. **(d,e)** Cells pretreated with ISL (10,20 μM) for 2 h before OGD/R, were then stained with Annexin V-PE/2-NDBG and detected by flow cytometry. **(f)** Detection of GLUT4 expression in H9C2 cells by immunofuorescence. Scale bar: 20 μm. **(g)** The representative western blot bands and quantitation of M-GLUT4, T-GLUT4, p-AKT and AKT in H9C2 cells. *p < 0.05, **p < 0.01, ***p < 0.001.

To test the function of ISL *in vitro*, firstly, we evaluated the cytotoxicity of ISL. CCK-8 assay was performed on H9C2 cells treated with various ISL concentrations (0, 2.5, 5, 10, 20, 40, and 80 μM) for 24 h. ISL treatment exhibited no significant cytotoxicity up to 80 μM (p = 0.0153, Figure 3a). According to previous study (Huang et al., 2020) and this results, 10 and 20 μM ISL were selected for further investigation. Next, we assessed the effect of ISL on OGD/R-induced injury in cardiomyocytes. Compared to the control, we investigated that OGD/R group significantly induced cell death (p < 0.0001). Conversely, ISL pretreatment increased cell viability after OGD/R injury (ISL-10+OGD/R *v. s.* OGD/R, p = 0.0122; ISL-20+OGD/R *v. s.* OGD/R, p = 0.0047; Figure 3b). In addition, ISL pretreatment significantly reduced OGD/R-induced LDH release (p < 0.0001, Figure 3c). To investigate whether this effect was associated with changes in glucose uptake, we used the fluorescent glucose analog 2-NBDG. OGD/R significantly inhibited 2-NBDG uptake (p = 0.0002), which was restored by ISL pretreatment (ISL-10+OGD/R *v. s.* OGD/R, p = 0.0087; ISL-20+OGD/R *v. s.* OGD/R, p = 0.0014; Figures 3d,e). In addition, immunofluorescence analysis revealed a significant decrease in GLUT4 protein expression in the cell membrane of H9C2 cells following OGD/R (Figure 3f). Similarly like *in vivo* assay, OGD/R resulted in decreased expression levels of p-AKT (p = 0.0008) and M-GLUT4 (p = 0.001), whereas ISL increased p-AKT (ISL-10+OGD/R *v. s.* OGD/R p = 0.0356; ISL-20+OGD/R *v. s.* OGD/R, p = 0.0052) and upregulated M-GLUT4 (ISL-10+OGD/R *v. s.* OGD/R, p = 0.011; ISL-20+OGD/R *v. s.* OGD/R, p = 0.0057) protein levels (Figure 3g).

### 3.4 Effect of IGFBP5 modulation on cardiac protection in H9C2 cells and the OGD/R model

To further investigate the potential targets of ISL in MIRI therapy. Using network pharmacology and docking methods, we found that ISL could directly interactions with IGFBP5. As shown in Figure 4a, the hydrogen bond formed by TYR70, LYS228 and ARG236 of IGFBP5 and ISL. With these interaction forces, the binding energy of protein-ligand complex was −6.8 kcal/mol, which exhibited a good performance. Then, we demonstrated a significant increase in IGFBP5 protein (Sham *v. s.* MI/R, p = 0.0054; Contorl *v. s.* OGD/R, p = 0.0037) and mRNA (Sham *v. s.* MI/R, p = 0.0018; Contorl *v. s.* OGD/R, p < 0.0001) expression levels in the MIRI group. However, ISL pretreatment reversed this increase, restoring expression levels to baseline (ISL-10+MI/R *v. s.* MI/R, p = 0.044; ISL-20+MI/R *v. s.* MI/R, p = 0.0299; Figure 4b. ISL-10+OGD/R *v. s.* OGD/R, p = 0.0256; ISL-20+OGD/R *v. s.* OGD/R, p = 0.0438; Figure 4c. ISL-10+MI/R *v. s.* MI/R, p = 0.0238; ISL-20+MI/R *v. s.* MI/R, p = 0.0302; Figure 4d. ISL-10+OGD/R *v. s.* OGD/R, p = 0.0006; ISL-20+OGD/R *v. s.* OGD/R, p = 0.0003; Figure 4e). To investigate the role of IGFBP5 in cardiac protection, lentiviral constructs were used to achieve knockdown and overexpression of IGFBP5 in H9C2 cells. Three pairs of IGFBP5-specific shRNA sequences and oe-IGFBP5 were designed, cloned with pLKO vectors, and subsequently transformed into competent cells. Western blot analysis confirmed successful manipulation of IGFBP5 protein expression 72 h post-transfection. IGFBP5 overexpression was achieved in the oe-IGFBP5 group (p = 0.0003), whereas IGFBP5-shRNA significantly suppressed IGFBP5 expression with sh-IGFBP5#2 and sh-IGFBP5#3 lentivirus (sh-Scramble *v. s.* sh-IGFBP5#1, p = 0.4211; sh-Scramble *v. s.* sh-IGFBP5#2, p = 0.0068; sh-Scramble *v. s.* sh-IGFBP5#3, p = 0.0047; Figures 4f,g), which were been selected to further study. Additionally, we evaluated the effects of IGFBP5 modulation on cell viability under OGD/R conditions using the CCK-8 assay. Our findings demonstrated a significant improvement in cell viability in the sh-IGFBP5 group. Conversely, the oe-IGFBP5 group exhibited a decrease in cell viability; this effect was partially mitigated by ISL pretreatment (Contorl *v. s.* OGD/R, p < 0.0001; sh-Scramble *v. s.* sh-IGFBP5#2, p = 0.0013; sh-Scramble *v. s.* sh-IGFBP5#3, p = 0.0004; oe-Scramble *v. s.* oe-IGFBP5, p = 0.0004; oe-IGFBP5 *v. s.* oe-IGFBP5+ISL, p = 0.0033; Figure 4h). Consistent with our findings, a significantly decreased LDH leakage rate was observed in the IGFBP5 silencing group, whereas the IGFBP5 overexpression group exhibited a significantly increased LDH leakage rate. ISL pretreatment mitigated the LDH leakage rate in the overexpression group (Contorl *v. s.* OGD/R, p < 0.0001; sh-Scramble *v. s.* sh-IGFBP5#2, p < 0.0001; sh-Scramble *v. s.* sh-IGFBP5#3, p = 0.002; oe-Scramble *v. s.* oe-IGFBP5, p = 0.0041; oe-IGFBP5 *v. s.* oe-IGFBP5+ISL, p = 0.0079; Figure 4i).

### 3.5 Effect of IGFBP5 modulation on cardiac protection in the MI/R rat model

**FIGURE 5:**
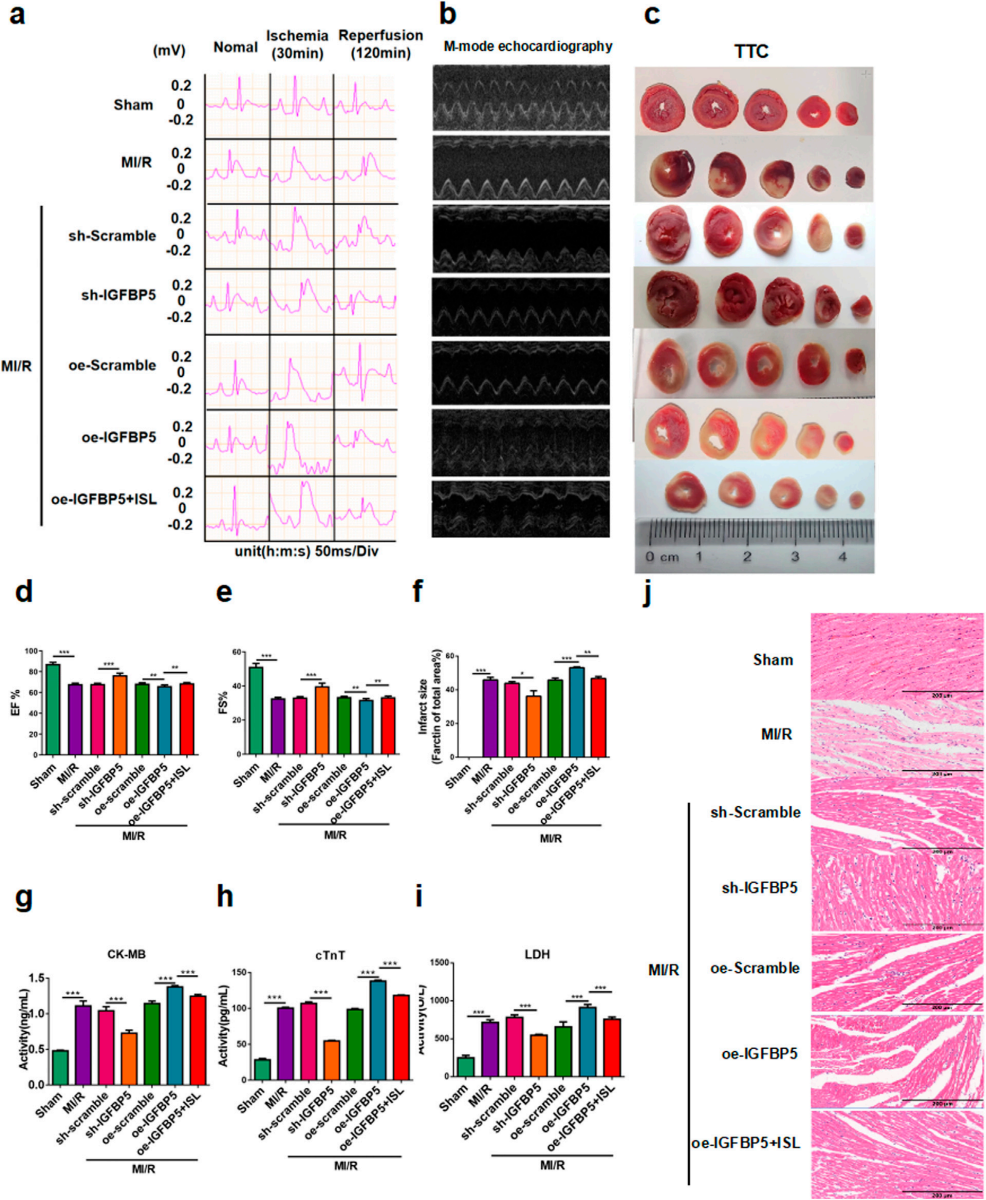
IGFBP5 promoted MI/R injury in rats. **(a)** Representative ECG traces in each group. **(b)** M-mode echocardiography performed in each group (n = 5). **(c)** TTC staining detected myocardial infarction area (n = 3). **(d,e)** The EF% and FS% of M-mode echocardiography. **(f)** The quantification of infarct size was displayed. **(g–i)** The levels of CK-MB, cTnT and LDH were detected (n = 5). **(j)** H&E staining of myocardial tissue during MI/R in rats. Scale bar: 200 μm *p < 0.05, ***p < 0.001.

To investigate the role of IGFBP5 in cardiac protection, adeno-associated virus were used to achieve silencing and overexpression of IGFBP5 in SD rats. Left ventricular M-mode echocardiography was assessed cardiac function after MIRI, and sh-IGFBP5 significantly attenuated MI/R-induced reductions in ejection fraction and fractional shortening. Conversely, the oe-IGFBP5 group exhibited a decrease in cardiac function; this effect was partially mitigated by ISL pretreatment (Figures 5b,d,e). Moreover, oe-IGFBP5 group exhibited a significantly larger infarct size compared to the MI/R group. However, ISL or sh-IGFBP5 significantly reduced infarct size compared to the MI/R group (Sham *v. s.* MI/R, p < 0.0001; sh-Scramble *v. s.* sh-IGFBP5, p = 0.0176; oe-Scramble *v. s.* oe-IGFBP5, p = 0.0007; oe-IGFBP5 *v. s.* oe-IGFBP5+ISL, p = 0.0011; Figures 5c,f). Serum levels of CK-MB, cTnT and LDH were significantly decreased in sh-IGFBP5 group and increased in oe-IGFBP5 group, compared with MI/R group (p < 0.0001, Figures 5g–i). H&E staining results showed the higher expression of IGFBP5, the more serious damaged to the myocardium, however, ISL treatment reduced this damage, indicating its cardiac-protective effect was related to IGFBP5 (Figure 5j).

### 3.6 Effect of IGFBP5 modulation on cardiac glucose metabolism via the AKT/GLUT4 pathway

**FIGURE 6:**
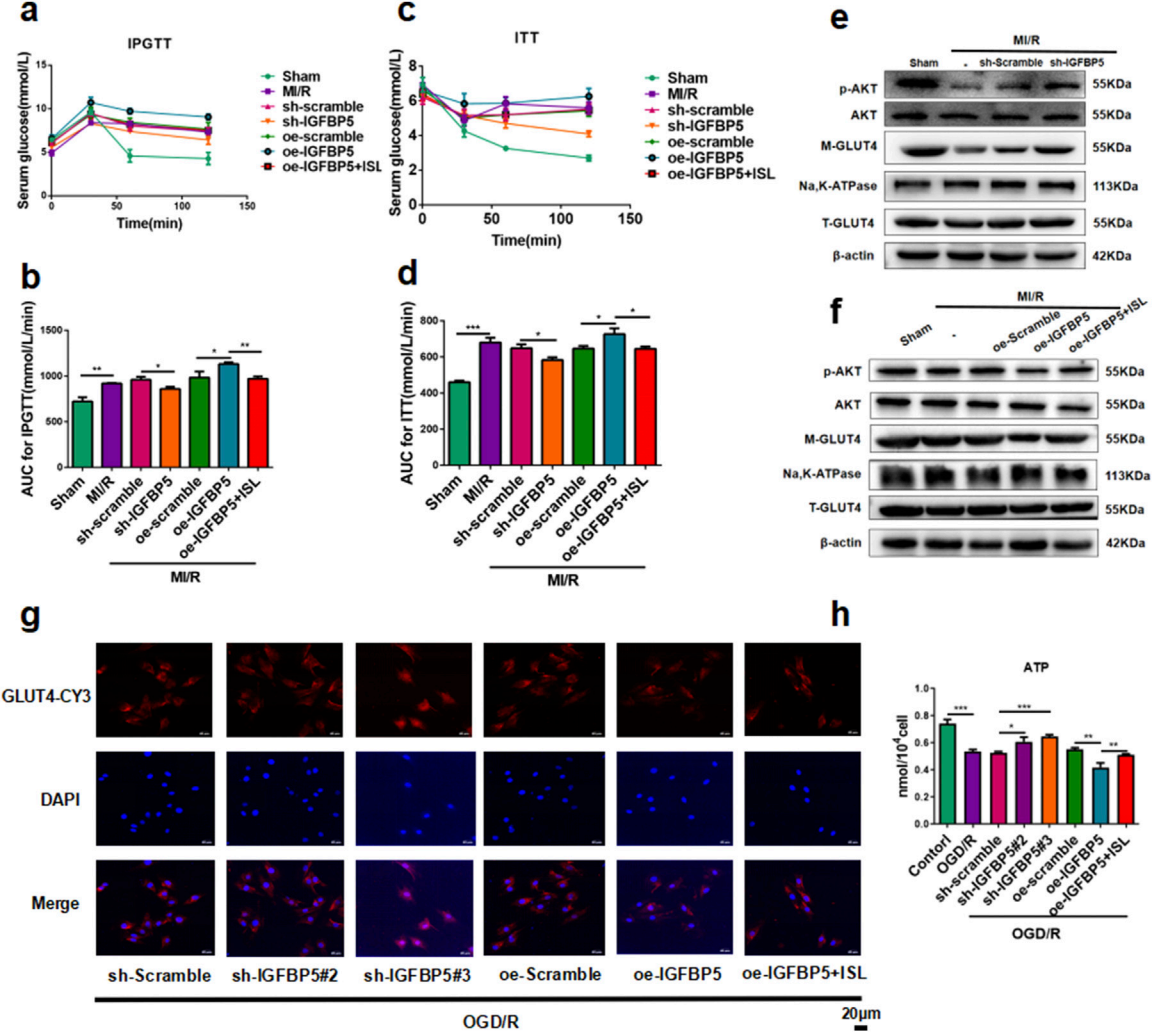
Effect of IGFBP5 modulation on cardiac glucose metabolism via the AKT/GLUT4 pathway in rats and H9C2 cells. **(a–d)** The glucose **(a,c)** or AUC **(b,dD)** results of IPGTT and ITT (n = 5). **(e,f)** The representative western blot bands and quantitation of M-GLUT4, T-GLUT4, p-AKT and AKT in SD-rats following IGFBP5 knockdown **(e)** or over-expression **(f)** (n = 3). **(g)** Detection of GLUT4 expression in H9C2 cells by immunofuorescence. Scale bar: 20 μm. **(h)** The levels of ATP were detected. *p < 0.05,**p < 0.01, ***p < 0.001.

To testify the role of IGFBP5 with IR, the IPGTT and ITT revealed significantly higher blood glucose levels and area under the concentration-time curve in the oe-IGFBP5 group compared to the MI/R group (IPGTT AUC, p = 0.0206; ITT AUC, p = 0.0183). Notably, pre-treatment with ISL or sh-IGFBP5 significantly improved these parameters, suggesting its potential role in regulating insulin sensitivity (IPGTT AUC, sh-Scramble *v. s.* sh-IGFBP5, p = 0.0107; oe-IGFBP5 *v. s.* oe-IGFBP5+ISL, p = 0.0013. ITT AUC, sh-Scramble *v. s.* sh-IGFBP5, p = 0.0127; oe-IGFBP5 *v. s.* oe-IGFBP5+ISL, p = 0.0165. Figures 6a–d). As shown in Figures 6e,f, compared with the OGD/R group, we investigated that sh-IGFBP5 exhibited an increase in AKT phosphorylation (p = 0.0174) and M-GLUT4 (p = 0.0002) protein levels, while the oe-IGFBP5 further reduced the expression levels of p-AKT (p = 0.0077) and M-GLUT4 (p = 0.0109). Notably, ISL pretreatment significantly increased the p-AKT (p = 0.0391) and M-GLUT4 (p = 0.0336) protein levels compared to the IGFBP5 overexpression group. Moreover, GLUT4 location in H9C2 cell membranes was significant increase in the sh-IGFBP5 group compared to the OGD/R group. Conversely, the overexpression group exhibited a further reduction in M-GLUT4, partially increased by ISL pretreatment (Figure 6g). The levels of ATP were significantly elevated in the sh-IGFBP5 group compared to the OGD/R group (sh-Scramble + OGD/R *v. s.* sh-IGFBP5#2+OGD/R, p = 0.0155; sh-Scramble + OGD/R *v. s.* sh-IGFBP5#3+OGD/R, p = 0.0002). ISL significantly reduced the further elevated markers of oe-IGFBP5 group, indicating its effect of energy metabolism to attenuate IR (oe-IGFBP5+OGD/R *v. s.* oe-IGFBP5+ISL + OGD/R, p = 0.0094; Figure 6h).

## 4 Discussion

Glucose metabolism is a critical determinant of energy production in cardiomyocytes; it includes the uptake, transformation, and utilization of glucose, serving as a key substrate for energy production. This intracellular pathway includes a series of well-defined steps, including glucose transport, glycolysis, glycogen synthesis, and breakdown, and the pentose phosphate pathway (Tran and Wang, 2019). Myocardial ischemia disrupts energy homeostasis in cardiomyocytes, leading to dysfunction. During this ischemic state, glycogen breakdown and glycolysis become the primary ATP production pathways (Zuurbier et al., 2020). However, reperfusion can lead to a paradoxical surge in glucose metabolism while restoring oxygen and nutrient supply. Although this pathway is crucial for replenishing cellular energy stores, excessive glucose utilization can result in the accumulation of detrimental metabolites, ultimately hindering ATP production and exacerbating MIRI (Lopaschuk and Stanley, 1997). Recent studies have revealed myocardial IR as a key driver of MIRI, primarily due to impaired glucose uptake and utilization caused by reduced GLUT4 translocation in cardiomyocytes (Liu et al., 2013); this is further supported by observations in non-diabetic patients undergoing coronary stent implantation for AMI. These patients exhibit a generalized increase in the HOMA-IR index, a marker of IR (Nishio et al., 2006; Farhan et al., 2021). This finding suggests that revascularization in the setting of damaged myocardium leads to significant IR; an increased IR index is an independent risk factor for restenosis after a coronary stent implantation (Kasem et al., 2021). Wang et al. (Wang et al., 2019) demonstrated that MIRI induces hyperglycemia and hyperinsulinemia, while simultaneously deactivating AKT signaling and disrupting GLUT4 translocation. These findings highlight the crucial role of glucose metabolism in MIRI pathogenesis. Therapeutic strategies that modulate glucose metabolic pathways hold promise for mitigating the severity of MIRI and protecting cardiomyocytes.

*Glycyrrhiza uralensis* Fisch., a commonly used Chinese herbal medicine with functions such as clearing heat, relieving cough, eliminating phlegm, relieving pain, and detoxifying (Yang et al., 2017), contains the chalcone compound, ISL. ISL exhibits anti-inflammatory and antioxidant properties, making it a popular choice for treating respiratory and liver ailments, such as non-alcoholic fatty liver disease and acute lung injury (Chen et al., 2021; Wen et al., 2023). It inhibits the NF-κB pathway, reduces oxidative stress, and eliminates inflammation. In addition, it stimulates the Nrf2 pathway in rat livers, reducing liver toxicity (Al-Qahtani et al., 2022). Several studies suggest ISL as an important functional component in licorice. The medicinal properties of compounds are generally evaluated based on factors, including physical and chemical properties, pharmacokinetics, tissue distribution, pharmacology, and toxicity (Jha et al., 2021). The relatively low molecular weight of ISL indicates its potential to penetrate cell membranes (Tian et al., 2021). This is further supported by studies demonstrating its distribution in the liver, kidney, heart, spleen, and lungs following intravenous administration at 20 mg/kg ^40^; this widespread distribution supports the potential therapeutic effects of ISL on various organ systems, including the liver, urinary tract, heart, and lungs.

ISL demonstrates promise as an anti-diabetic agent, having been identified as an aldose reductase inhibitor as early as 1990 (Aida et al., 1990; Gaur et al., 2014). A recent study demonstrated that ISL can restore metabolic homeostasis in diabetes mice fed a high-fat, high-glucose diet; this effect included improved insulin signaling sensitivity, promotion of liver glycogenesis, and inhibition of liver adipogenesis. ISL exerts its anti-diabetic effect through mechanisms involving activation of the AMPK signaling pathway and inhibition of mTORC1, both of which are key regulators of cellular metabolism (Yang et al., 2022). These mechanisms potentially contribute to its effectiveness in treating complications associated with type 2 diabetes mellitus, such as nephropathy (Alzahrani et al., 2020a; Sun et al., 2021), neuropathy (Yerra et al., 2017), aortic injury (Alzahrani et al., 2021), retinopathy (Alzahrani et al., 2020b), and myocarditis (Gu et al., 2020). In this study, we established an *in vivo* rat model and *in vitro* OGD/R-induced H9C2 cells model of MIRI, resulting in significant myocardial damage and increased IR levels. ISL pretreatment considerably reduced myocardial IR and improved tissue integrity.

Using network pharmacology and docking methods, we investigated that the potential target of ISL in MIRI therapy. Our study showed that ISL have three binding sites, Arg236, Tyr70, and Lys228, with IGFBP5. Nowadays, seven IGF-binding proteins (IGFBPs) have been identified, which mainly participated to glucose metabolism and insulin function (Shen et al., 2024). Then, RT-qPCR was performed to evaluate IGFBPs mRNA expression levels in *in vivo* and *in vitro* models. Only IGFBP5 was higher expression in MIRI rat and OGD/R cells, compared with match normal group (data not shown). IGFBP5, a protein abundantly expressed in normal and cancerous tissues, regulates tissue and cell growth and development. Multiple studies suggest a proapoptotic role for IGFBP5 in cardiomyocytes (Leung et al., 2014). High IGFBP5 levels have been associated with the initiation and progression of heart disease (Fischer et al., 2004; Song et al., 2013). Notably, IGFBP5 possesses a greater binding affinity for insulin-like growth factor (IGF) compared to IGF receptors, thereby regulating IGF bioavailability (Lai et al., 2020). The IGF signaling pathway plays a critical role in cardiac development and regeneration by promoting the differentiation and proliferation of cardiac progenitor cells (Bowman et al., 2010; Huang et al., 2013; Li et al., 2011). Furthermore, due to the significant homology between insulin and IGF receptors, along with the shared downstream signaling molecules, IGF plays a crucial role in maintaining glucose homeostasis (Rachdaoui, 2020). In order to confirm the function of IGFBP5 in MIRI, using lentivirus to silencing IGFBP5, we testified that knockdown IGFBP5 mitigated MIRI and myocardial damage. Conversely, IGFBP5 overexpression (OE-IGFBP5) exacerbated these effects. Notably, the administration of ISL attenuated the additional damage caused by IGFBP5 overexpression both *in vivo* and *in vitro*. In the traditional IGF signaling pathway, IGFBP is mainly involved in the binding effect of IGF and IGF1R. We therefore also examined the activation of the IGF signaling pathway, and we found that ISL or IGFBP5 knocked down promoted the phosphorylation of IGF1R compared to the MI/R or OGD/R groups (Supplementary Figure S1). Therefore, ISL may interfere with AKT phosphorylation by interfering with IGFBP5/IGF1R signaling pathway, and this part of the experiment still needs to be explored in the future.

In summary, these findings suggest that IGFBP5 mediated the effectiveness of ISL, which promoted the detrimental effects of MIRI via the AKT/GLUT4 pathway. Consequently, ISL represents a potential therapeutic candidate for myocardial injury; the IGFBP5/AKT/GLUT4 pathway may be a viable therapeutic target for MIRI.

## 5 Conclusion

Our study suggests that ISL, a key flavonoid from *Glycyrrhiza*, may offer cardio-protective effects by ameliorating myocardial IR through the IGFBP5/AKT/GLUT4 pathway (**Graphical abstract**). These findings offer a compelling rationale for further investigation of the therapeutic potential of ISL in mitigating MIRI.

## Data Availability

The original contributions presented in the study are included in the article/Supplementary Material, further inquiries can be directed to the corresponding authors.
