# Supplementary material for: IGFBP5 mediates the therapeutic effect of isoliquiritigenin in myocardial ischemia-reperfusion injury via AKT/GLUT4 regulated insulin resistance
Source: Front Pharmacol. 2025 Apr 29;16:1544869. doi: 10.3389/fphar.2025.1544869 (PMC12069378; doi:10.3389/fphar.2025.1544869)
Supplement: Supplementary file 2 [file DataSheet1.docx]

**Supplementary Figure**

**Materials**

The antibody of p-IGF-1R (1:1000, #9750, CST), IGF-1R (1:1000, #3024, CST) were purchased in Cell Signaling Technology, Inc.

**Results**


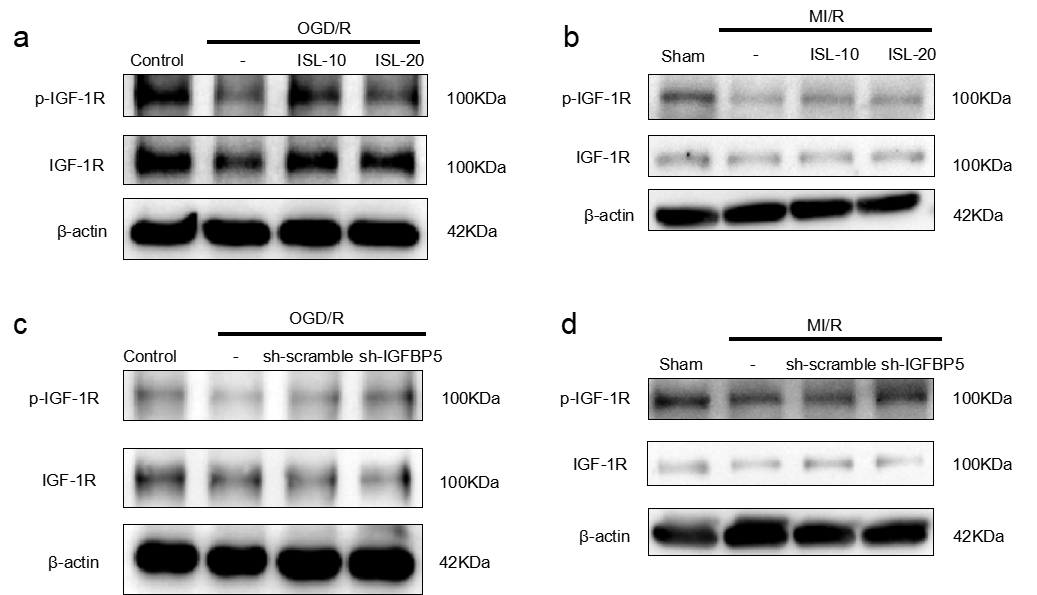


Supplementary Figure1. ISL or knockdown IGFBP5 promoted IGF-1R activation.

(a-d) The protein expression levels of p-IGF-1R or IGF-1R in heart tissues and H9C2 cells after treatment of ISL or knockdown IGFBP5.
